# Brain-dead Donation Rate in Month of Ramadan and the other Months: 2005-2014

**Published:** 2017-08-01

**Authors:** M. Aghighi, M. Mahdavi-Mazdeh, M. Saberi Isfeedvajani, S. A. Tavakoli, N. Tirgar, A. Heidary Rouchi

**Affiliations:** 1 Iranian Tissue Bank and Research Center, Tehran University of Medical Sciences, Tehran, Iran; 2 Department of Community Medicine, Faculty of Medicine, Baqiyatallah University of Medical Sciences, Tehran, Iran

**Keywords:** Organ donation, Brain death, Religion and medicine, Islam, Consent

## Abstract

Regardless of the level of development, religion and beliefs have crucial impact on people’s attitude towards organ donation. Although organ donation in Islam is obviously appraised, mainly due to lack of an appropriate infrastructure, post-mortem donation rate in Islamic countries is not comparable to successful settings. We conducted this study to assess the extent of contribution of factors that reduce the level of effectiveness, and also to determine the impact of altruistic feelings in the month of Ramadan on family refusal as the leading modifiable contributor to organ donation rate. All records of potential and actual brain-dead donors, referred to Organ Procurement Unit of the Iranian Tissue Bank, from January 1, 2005 to December 31, 2014, were analyzed. In each year, the number of potential and actual donors in the month of Ramadan was compared to the mean value in the remaining 11 months. Of 1758 total potential donors in 10 years, 464 cases became actual donors (26.4% as overall level of effectiveness). The reasons for non-effectiveness were medical contraindications (25.4%), cardiac arrest before referral or during maintenance (7.4%), family refusal (30.8%), judicial refusal (8.7%), *etc* (1.3%). Analysis showed no significant differences between donation rates (both potential and actual) in Ramadan and non-Ramadan months for potential (Δ=3.55, 95% CI: -6.7 to 13.8) and actual donors (Δ=1.35, 95% CI: -2.3 to 5). Despite the undeniable role of religion and beliefs in the establishment of organ procurement program from brain-dead donors, there was no monthly variability in post-mortem organ donation rate.

## INTRODUCTION

Religious concerns and cultural values are hardly resolvable barriers to set up organ donation and transplantation program from deceased donors [1, 2]. Organ transplantation is an unavoidable need for patients suffering from end-stage organ failure. A number of patients die every day awaiting organ transplantation [3]. In different settings with running practices, various approaches have been adopted to increase donation rate; nevertheless, the results have not yet been satisfactory [4, 5]. Almost in all faiths, saving life is a common value. In Islam, in particular, organ donation, as a life-saving and altruistic behavior, is clearly appraised. It is stated in Quran that “whoever saves the life of someone, it is as he saved mankind entirely” (Sura 5, Aya 32). Notwithstanding, due to other reasons, the post-mortem organ donation and transplantation in Islamic countries has not yet been as successful as other experiences [6-8].

In the context of organ donation, different people have different views, even with identical religion. So, different indices are role-players and therefore should be taken into consideration. Infrastructure, type of consent and people’s positive attitude are widely known contributors; however, the importance of these parameters has not yet been determined [9]. To realize how important these factors are, could lead transplant coordinators to focus mainly on effective factors to increase the proportion of actual to potential donors, as the level of effectiveness.

From Muslims’ point of view, Ramadan is a month of grace in which Allah grants Muslims peace and forgiveness and the doors of blessing would be open (The holy Quran, Sura 2, Aya 185). It is why the people plead more sincerely divine and infinite mercy for their beloved brain-dead to be miraculously revived. We therefore conducted this study to assess the potential impact of altruistic feelings in the month of Ramadan on organ donation rate.

## MATERIALS AND METHODS

Iranian Tissue Bank, affiliated to Tehran University of Medical Sciences, Iran, has a running organ procurement unit (OPU) from 2000. All records of potential and actual brain-dead donors, referred to this unit, from January 1, 2005 to the December 31, 2014, were analyzed. In each year, the number of potential and actual donors in the month of Ramadan was compared to the mean value in remaining 11 months. The overall level of effectiveness (proportion of potential donors who become actual donors and donate solid organ(s) for transplantation), the reasons of non-effectiveness, and the percentage of donated organs were other variables of interest. The number of potential and actual donors in Ramadan and non-Ramadan months in 10 years was compared as two independent groups. Statistical analysis was conducted using SPSS/PASW ver 18 (IBM SPSS Statistics, SPSS Inc, Chicago, IL, USA).

## RESULTS

The mean±SD age of actual donors during the study period was 29.6±13.3 years. Male/female ratio was 2.1. All deceased organ donors were heart-beating brain-dead donors. The main causes of brain death were cranio-cerebral trauma due to traffic accident (56.6%) and cerebrovascular accident (18.9%). Of 1758 total potential donors in 10 years, 464 cases became actual donors (26.4% as the overall level of effectiveness). The level of effectiveness increased from 8.3% in 2005 to 41% in 2014. The mean implanted organs per donor increased from 2.7 in 2005 to 3.6 in 2014. The reasons for non-effectiveness were medical contraindications (25.4%), cardiac arrest before referral or during maintenance (7.4%), family refusal (30.8%), judicial refusal (8.7%), etc (1.3%). The proportion of suitable kidneys, liver, heart, lung, and pancreas for transplantation was 94%, 89%, 81%, 14%, and 93%, respectively. In addition to other variables, analysis showed no significant differences between donation rates (both potential and actual) in Ramadan and non-Ramadan months for potential (Δ=3.55, 95% CI: -6.7 to 13.8) and actual donors (Δ=1.35, 95% CI: -2.3 to 5). 

## DISCUSSION

The findings suggest that despite common presumed idea, there was no significant difference between donation rate in Ramadan and non-Ramadan months. The evidence suggests that despite the role of important predictors, religion and beliefs, in the field of organ donation, nothing replaces the importance of increased awareness, transparency, trust between medical staff and deceased’s families, and more importantly, systematic approach to this vital and absolutely scientific issue. At the meantime, to believe in “miracle” in religious events can inversely diminish the consent rate and counterbalancing the increased feeling of generosity [10]. Trained, updated, and dedicated professionals coordinated in a well-established team would be more productive than to rely on merely spiritual values. It seems, as well, that the increased people’s tendency to donate organs after death reflected from the increased number of issued donation cards in a period cannot necessarily be translated into the increased consent rate in special occasions such as Holy month of Ramadan [11, 12].

Ozer, *et* *al* (2010), gave evidences that according to the study in Turkey on 367 religious scholars, 88.2% stated that organ donation is appraised but just 1.4% of them had willingness to organ donation after death [13]. In another study in Turkey, Kececioglu, *et al*, showed that 26.2% of family refusal rate was due to religious beliefs [14]. 

In conclusion, to approach the families to offer the option of donation requires knowledge, experience, and professionalism. In this approach, all family concerns should be addressed and properly responded. Despite the undeniable importance of spiritual values in this context, the systematic approach to obtain consent and to create an ambience of trust are strategies with highest effectiveness [15-18].
